# The role of acupuncture in treating premature ejaculation and its probable neurobiological mechanism

**DOI:** 10.1186/s12610-024-00239-w

**Published:** 2024-12-12

**Authors:** Anmin Wang, Hao Wang, Dongyue Ma, Hongyuan Chang, Ziwei Zhao, Dicheng Luo, Fu Wang

**Affiliations:** Department of Andrology, Xiyuan Hospital of China Academy of Chinese Medical Sciences, Beijing, 100091 China

**Keywords:** Acupuncture, Premature ejaculation, Mechanism, Traditional Chinese medicine, Acupuncture, Éjaculation précoce, Mécanismes, Médecine traditionnelle chinoise

## Abstract

**Background:**

Premature ejaculation (PE) is one of the most common diseases in andrology and leads to serious male sexual dysfunction. Although several targeted oral drug therapies are used to treat PE, they often face challenges related to imprecise targeting and adverse effects. Acupuncture has shown potential in prolonging ejaculation time and improving couples' sexual quality of life. This review aims to summarize the benefits of acupuncture in treating PE and explore its probable neurobiological mechanisms.

**Results:**

The review included eight clinical trials involving 679 patients, of which 294 were treated with acupuncture. Furthermore, this review analyzed acupuncture points, needle retention time, treatment duration, and their probable neurobiological mechanisms. The proposed mechanisms include stimulating the frontal functional lobe, inhibiting spinal cord neural pathways, regulating serotonin levels, enhancing 5-hydroxytryptamine receptor IB excitability, reducing penile sensitivity, and modulating hormone levels.

**Conclusions:**

Acupuncture is a viable alternative or complementary therapy for PE, and neurobiological mechanisms appear to play a key role, but further experimental validation is needed.

## Résumé

**Contexte:**

L’éjaculation précoce (EP) est l’une des maladies les plus courantes en andrologie et entraîne un grave dysfonctionnement sexuel masculin. Bien que plusieurs traitements médicamenteux oraux ciblés soient utilisés pour traiter l’EP, ils sont souvent confrontés à des défis liés à un ciblage imprécis et à des effets indésirables. L’acupuncture a montré son potentiel à prolonger le temps d’éjaculation et à améliorer la qualité de vie sexuelle des couples. Cette revue vise à résumer les avantages de l’acupuncture dans le traitement de l’EP et à explorer ses probables mécanismes neurobiologiques.

**Résultats:**

La revue comprend huit essais cliniques impliquant 679 patients, dont 294 ont été traités par acupuncture. De plus, cette revue a analysé les points d’acupuncture, le temps de rétention de l’aiguille, la durée du traitement, et leurs probables mécanismes neurobiologiques. Les mécanismes proposés comprennent la stimulation du lobe fonctionnel frontal, l’inhibition des voies neuronales de la moelle épinière, la régulation des niveaux de sérotonine, l’amélioration de l’excitabilité du récepteur IB de la 5-hydroxytryptamine, la réduction de la sensibilité du pénis, et la modulation des niveaux d’hormones.

**Conclusions:**

L’acupuncture est une alternative viable ou une thérapie complémentaire de la prise en charge de l’EP, et les mécanismes neurobiologiques semblent jouer un rôle clé. Mais une validation expérimentale supplémentaire est nécessaire.

## Introduction

Premature ejaculation (PE) is the most common male sexual dysfunction, and currently, about 30% of men worldwide suffer from PE, but there is no consensus in the medical community on the definition of premature ejaculation [1, 2]. Schapiro et al. [3] categorize PE as either lifelong, where ejaculation occurs from the first sexual intercourse, about 1 min after the penis enters the vagina, or even before sexual intercourse; or acquired, where ejaculation latency is previously normal, but there is a significant shortening of ejaculation latency, usually less than 3 min. The etiology of PE is very complex, and the International Society for Sexual Medicine (ISSM) in 2014 proposed that it must include three elements: (i)a short ejaculatory latency;(ii) a perceived lack of control or inability to delay ejaculation;(iii) distress and interpersonal difficulty to the individual and/or partner (related to the ejaculatory dysfunction) [4].

Ejaculation consists of two processes: semen secretion and ejaculation [5]. Semen secretion is mainly innervated by adrenergic sympathetic nerves originating from the thoracolumbar (T10 ~ L2), while ejaculation is mainly accomplished with the cooperation of parasympathetic nerves and perineal nerves (somatic nerves) in the sacral medulla (S2 ~ S4). Some impulses from the cortex can also be transmitted directly to the ejaculatory centers. When the cortex is activated to a certain degree, the impulses are transmitted to the sympathetic chain through the brainstem and the anterior lateral columns of the spinal cord [6].

PE not only leads to a decline in the quality of sexual activities between spouses but also affects the relationship between couples, and even leads to anxiety, depression, and other adverse emotions [7]. Western drug treatments primarily involve the use of selective serotonin reuptake inhibitors (SSRIs), clomipramine, local anesthetics, dapoxetine, and tramadol. Among these, the oral medications paroxetine and dapoxetine have demonstrated greater effectiveness in treating premature ejaculation and have been well-utilized in clinical practice [8]. However, a Korean study revealed that some patients experienced adverse reactions, such as nausea, vomiting, and dizziness after taking dapoxetine [9], and as a result, the desired therapeutic effect was not achieved. Recently, traditional Chinese medicine (TCM) has been used in PE and has confirmed its efficacy and safety in this disease [10].

Acupuncture is a crucial component of TCM. In recent years, acupuncture has gradually gained recognition as an alternative therapy among many scholars and Western clinicians and it has been frequently used in treating various urological disorders, including sexual dysfunction in both males and females, but most studies were focused on erectile dysfunction (ED) [11–14]. Intriguingly, Zucker et al. suggest that most men with PE do not receive medication [15], implying that they may opt for acupuncture as a non-pharmacologic therapy. A Meta-Analysis has discussed the effectiveness and safety of acupuncture in the treatment of PE, however, they lacked further exploration of the mechanisms. In this review, we describe the main effects and mechanisms of acupuncture for PE, such as the acupoint selection, time of needle retention, and duration of needling. We sincerely hope that this paper will present a new perspective on the treatment of PE.

## Materials and methods

### Search strategy

Four English databases (PubMed, Embase, Web of Science, and Cochrane Library) and four Chinese databases (China National Knowledge Infrastructure, China Science and Technology Journal Database, Wanfang Data, Sinomed) were systematically searched for relevant studies (up to October 2023). Additionally, we reviewed the reference lists of studies identified through our search strategy and selected studies that appeared to be relevant based on keywords. The following search terms were used: acupuncture, acupuncture treatment, electroacupuncture, acupuncture therapy, fire needling, scalp acupuncture, ear acupuncture, abdominal acupuncture, warming acupuncture, premature ejaculation, ejaculation, ejaculatio praecox, early ejaculation, rapid ejaculation, clinical trial, clinical article, clinical study, controlled study, and randomized controlled trial. All searches were conducted using a combination of subject headings and free words adapted to specific databases. This review included only articles written in English and Chinese. The search strategies are detailed in Table 1.

**Table 1:** PubMed search strategy

No	Search item
#1	Acupuncture [Title/Abstract]
#2	Acupuncture treatment [Title/Abstract]
#3	Electroacupuncture [Title/Abstract]
#4	Acupuncture therapy [Title/Abstract]
#5	Fire needling [Title/Abstract]
#6	Scalp acupuncture [Title/Abstract]
#7	Ear acupuncture [Title/Abstract]
#8	Abdominal acupuncture [Title/Abstract]
#9	Warm acupuncture [Title/Abstract]
#10	Or/#1-#9
#11	Premature ejaculation [Title/Abstract]
#12	Ejaculation [Title/Abstract]
#13	Ejaculatio praecox [Title/Abstract]
#14	Early ejaculation [Title/Abstract]
#15	Rapid ejaculation [Title/Abstract]
#16	Or/#11-#15
#17	Clinical trial [Publication Type]
#18	Clinical article [Publication Type]
#19	Clinical study [Publication Type]
#20	Controlled study [Publication Type]
#21	Randomized controlled trial [Publication Type]
#22	Or/#17-#21
#23	#10 and #16 and #22

### Inclusion criteria

#### Type of study

The study types were clinical trials that take acupuncture as the primary treatment for PE, and the document language is limited to English or Chinese.

#### Type of participants

Patients were men over 18 years of age who met the diagnostic criteria for PE [16–18], whether it is lifelong or acquired.

#### Type of intervention

Common acupuncture (including needling, electroacupuncture, fire acupuncture, scalp acupuncture, auricular acupuncture, abdominal acupuncture, and warm acupuncture) was used either alone or in combination with other treatments, regardless of the location of the acupuncture points, frequency of treatment, and duration of treatment, there was no need for a control group.

#### Type of comparisons

The control group received conventional treatment, drug treatment, placebo acupuncture, bogus acupuncture or no treatment, or no control group.

#### Type of outcomes

The principal outcome indicator is the intravaginal ejaculation latency time (IELT [19]), which is the time between the insertion of the penis into the vagina and the moment when it begins to ejaculate. Secondary outcome indicators are the Premature Effusion Diagnostic Tool(PEDT [20]: The PEDT consists of 5 questions, control of ejaculation, frequency of premature ejaculation, minimal sexual stimulation, stress, and interpersonal difficulties due to premature ejaculation. Each question has 5 different levels of options on a scale of 0 to 4, for a total score of 20. A score of ≤ 8 was considered no premature ejaculation, 9 to 10 possible premature ejaculation, and ≥ 11 premature ejaculation), the Chinese Index of Premature Ejaculation–5 (CIPE-5 [21]: The Chinese Index of Premature Ejaculation–5 consists of 10 questions, including ejaculation latency, ease of ejaculation control, patient's satisfaction with sex life, spouse's satisfaction with sex life, and patient's anxiety and tension about sex life. Each question has 5 different levels of options, with a total score of 25, with higher scores indicating a lower level of premature ejaculation), serum testosterone level, and adverse reactions.

## Exclusion criteria

1. Patients with PE for other known reasons (such as prostatitis, erectile dysfunction, urethritis, severe central nervous system damage, or those who took medications that may cause PE), and with a history of other treatments within three months.
2. Nonclinical studies, reviews, animal experiments, case series, case reports, conference papers, and cross-over studies.
3. Non-acupuncture therapy, including moxibustion, shuttle moxibustion, acupoint application, and massage of the related acupoints.
4. Duplicate publications and trials with incomplete data.

### Data collection and analysis

#### Selection of studies

Two authors (Anmin Wang and Hao Wang) searched for articles based on the outline search strategy and sum up the findings. Duplicates were excluded, some articles were excluded after analyzing the content of the title and abstract, and those that did not meet the inclusion and exclusion criteria were excluded after analyzing the full text. Throughout the process, dissenting literature was resolved via discussions or referred to another author (Dongyue Ma) for adjudication.

#### Data extraction

All articles were read independently by two authors (Anmin Wang and Hao Wang) and data were extracted from these articles according to previously defined criteria. These data are organized in standardized tables by author, year of publication, age range of participants, sample size, acupoints, duration, control intervention, and outcome indicators.

## Result

**Fig. 1:**
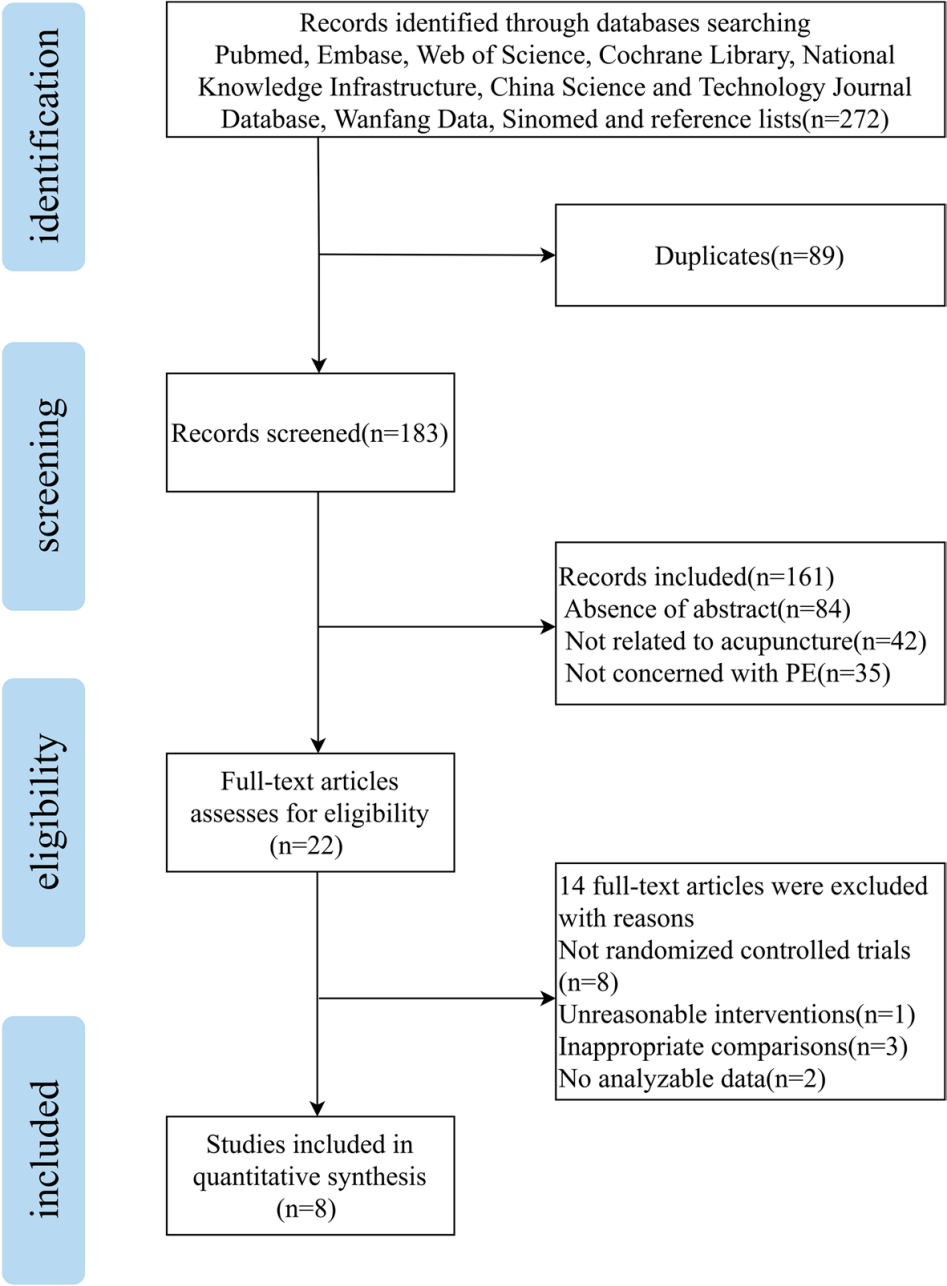
Flow chart of the study search

Following the screening process described above, we extracted a total of 272 documents from the databases. After excluding 89 duplicates and evaluating titles and abstracts, 161 cases were further excluded. Following a thorough reading of the entire text, 8 cases of literature were excluded because they did not meet the criteria of randomized controlled trials [22–29], 1 case was removed due to unreasonable interventions [30], 3 cases were denied inclusion because of inappropriate comparisons [31–33], and 2 cases were dismissed as no analyzable data could be obtained, even after attempting to contact the original authors [34, 35]. Thus, we finally included 8 papers, including 3 in English [36–38] and 5 in Chinese [39–43], as shown in Fig. 1.

The eight studies included in this review were all randomized controlled trials involving 679 patients with premature ejaculation, of which 294 received acupuncture, electro-acupuncture, or warm acupuncture. The remaining participants were administered SSRIs, herbal tonics, sham acupuncture, or drug-combination acupuncture. These studies employed coordinated multi-acupoint treatment to enhance clinical efficacy, mentioning a total of 27 acupoints. The selected acupoints used the most frequently were SanYinjiao (SP6), Zhongji (RN3), Taichong (LR3), and Guanyuan (RN4). The primary acupoints were most intimately associated with the foot-taiyin spleen meridian, the foot-jueyin liver meridian, and the ren meridian among the fourteen meridians, and the acupoints applied were mainly centered on the lower limbs and the abdomen. In addition, the needling duration varied among these studies, with most of them maintaining basic needle retention for 20 or 30 min. The course of treatment spanned 4 weeks in six studies, while two studies by He [43] and Li [41] extended the treatment duration to 8 weeks.

Although these eight studies presented variations in acupoints and duration of needle retention, they shared commonalities in terms of the outcome indicators. All of these randomized controlled trials primarily employed IELT as the key metric to determine the efficacy of acupuncture or drug therapy. Results from seven studies indicated a prolonged IELT post-treatment, whether in the acupuncture group or the combined acupuncture and medicine group, with the combination approach demonstrating superior performance. Although the acupuncture group wasn't as effective as the SSRIs, it significantly delayed ejaculation time relative to the sham acupuncture or placebo group. The remaining study by He [43] used therapy efficiency as the unique indicator, defined as prolonged ejaculation time and spouse satisfaction within six months of follow-up, inherently aligning with the International Index of Erectile Function (IIEF). Additionally, three studies [36, 38, 42] used the PEDT score as the secondary indicator of PE, and two studies [39, 41] employed the CIPE-5 score as the secondary indicator, with effective changes in the acupuncture group surpassing those in the sham-acupuncture or placebo group. Lu [37] and Tang [40] included serum testosterone levels as an outcome indicator, noting reductions across all groups post-treatment, suggesting the potential of acupuncture or electroacupuncture to improve premature ejaculation symptoms through serum testosterone regulation. Notably, four studies [37, 39, 40, 43] used sexual life satisfaction of either the spouse or patient as a judgment indicator, with increased satisfaction following acupuncture in these researches.

A total of 4 out of all these studies reported adverse events during acupuncture. Lu [37] reported 5 cases of adverse reactions in the acupuncture group, including 2 cases of dizziness and 3 cases of subcutaneous hematoma. Tang [40] also reported dizziness, subcutaneous hematoma, and stomach discomfort, however, these adverse reactions disappeared after treatment. Li's study [41] documented 4 cases of adverse reactions, but he did not provide further elaboration. Only one case of adverse reactions to body fatigue in Feng's [39] study. In the remaining four studies [36, 38, 42, 43], either no adverse events occurred during acupuncture or they were not mentioned. (Table 2) Therefore, it is crucial to weigh the risks and benefits when developing a treatment plan. Although SSRIs are commonly used in the treatment of PE and have been shown to significantly prolong ejaculation time, their use comes with several side effects. According to Bala et al. [44], post-SSRI sexual dysfunction (PSSD) is a condition in which patients continue to have sexual side effects after discontinuation of SSRI use. Common symptoms include genital anesthesia, pleasure-less or weak orgasm, decreased sex drive, erectile dysfunction, and premature ejaculation, for which there are no definitive treatments and which severely impact patients' quality of life and overall sexual satisfaction. In contrast, the side effects of acupuncture are relatively mild and short-lived. While acupuncture may be slightly inferior to SSRIs in terms of prolonging ejaculation, it offers a treatment option with fewer and milder long-term side effects. This makes acupuncture a viable alternative or complementary therapy for the treatment of PE, especially for patients concerned about the long-term adverse effects of SSRIs.

**Table 2:** Summary of the included studies

References	Number of men	Age(year)	Acupoints	Intervention	Outcomes	Adverse Reactions
Sahin et al (2016) [36]	120	T:34.1 ± 6.5 C_1_:33.2 ± 6.5 C_2_:32.9 ± 5.0 C_3_:33.7 ± 7.0	BL30 Baihuanshu, BL52 Zhishi, ST36 Zusanli, LI4 Hegu, LR3 Taichong, EX-HN3 Yintang, CV3 Zhongji	AC, 20 min 2/week for 4 weeks	IELT at 4 weeks for the acupuncture group was significantly higher than pre-treatment (*P* < 0.001); PEDT score also decreased in the acupuncture group compared to pre-treatment (*P* < 0.001); and the changes in the acupuncture group were significantly higher than the sham acupuncture group (*P* < 0.001)	None
Lu et al. [37]	50	T:32.7 ± 4.8 C:33.6 ± 5.0	CV3 Zhong ji, SP6 San Yinjiao	EA, 30 min 6/week for 4 weeks	IELT 3.28 ± 0.59(the electroacupuncture group) vs 3.09 ± 0.62(the control group), *P* < 0.05; sexual life satisfaction score of spouse 10.86 ± 1.49(pre-treatment) vs 4.85 ± 0.98(post-treatment), *P* < 0.05; testosterone level 13.28 ± 3.15(pre-treatment) vs 26.16 ± 5.26(post-treatment), *P* < 0.05; all the results were significantly higher than the control group, *P* < 0.05	Two cases of dizziness, and three cases of subcutaneous hematoma
Sunay et al (2011) [38]	90	T: 37.5 C_1_:40.4 C_2_: 38.3	ST36 Zusanli, LI4 Hegu, KI3Taixi, LR3Taichong, EX-HN3 Yintang, CV3 Zhongji	AC, 20 min 2/week for 4 weeks	Median PEDT score of acupuncture, and placebo groups were 16.0 and 15.5 before treatment, 11.0 and 16.0 after treatment (*P* = 0.001, and *P* = 0.314); Increase of IELTs with acupuncture was 65.7, *P* = 0.001	None
Feng et al (2021) [39]	68	T:33.4 ± 7.3 C:32.0 ± 6.1	ST36 Zusanli, SP6 Sanyinjiao, BL23 Shenshu, BL20 Pishu, DU4 Mingmen, DU3 Yaoyangguan, SP9 Yinlingquan	WA, 30 min 2/week for 4 weeks	IELT, CIPE-5 score and sexual life satisfaction of spouse in warm acupuncture group all improved after treatment: the treatment group was 106.15 ± 4.016, 19.97 ± 2.758 and 44.06, *P* < 0.01; before the treatment was 45.03 ± 3.316, 9.35 ± 2.533 and 34.21, *P* < 0.01	One case of fatigue
Tang et al (2016) [40]	120	NA	RN4 Guanyuan, RN3 Zhongji, LR2 Xingjian, GB4 Xiaxi, SP6 Sanyinjiao, BL18 Ganshu, BL32 Ciliao, BL28 Bladder Shu	AC, 30 min 5/week for 4 weeks	IELT in the group containing acupuncture was prolonged after treatment(*P* < 0.05); sexual life satisfaction score of spouse in acupuncture and drug co-administration group also increased than pre-trentment (*P* < 0.05); the testosterone level decreased in the group containing acupuncture after treatment (*P* < 0.05)	Three case of dizziness during acupuncture or injection,five cases of subcutaneous hematoma, four cases of stomach discomfort,all adverse reactions disappeared after treatment
Li et al (2015) [41]	69	T: 28.0 ± 4.4 C_1_: 28.0 ± 4.6 C_2_: 23 ± 4.2	BL23 Shenshu, RN6 Qihai, RN4 Guanyuan, SP6 Sanyinjiao, KI1Yongquan	EPAS, 4 min/acupoint/time, 3/week for 8 weeks	IELT of the group containing EPAS had markedly increased (0.92 ± 0.12 vs 2.78 ± 0.17, *P* < 0.05; 0.91 ± 0.09 vs 5.31 ± 0.13, *P* < 0. 01); CIPE-5 score decreased (12.8 ± 2.9 vs 19.5 ± 1.9, *P* < 0.05; 13.1 ± 2.8 vs 25.2 ± 2.1, *P* < 0.01); the effectiveness rate was 47.8% in the simple EPAS group	A total of 4 cases were seen adverse reactions, but it was not elaborated
Wang et al (2013) [42]	60	T: 33.1 ± 4.3 C_1_: 32.5 ± 4.3 C_2_: 32.4 ± 3.9	LR3 Taichong, KI3 Taixi, SP6 Sanyinjiao, RN4 Guanyuan, DU20 Baihui	AC, 20 min 3/week for 4 weeks	IELT had prolonged in the acupuncture group compared to pre-treatment (*P* < 0.001); PEDT score also decreased in the acupuncture group (*P* < 0.001),but no significant differences were found between the PEDT score of the paroxetine and the acupuncture group (*P* = 0. 07 > 0.05)	None
He et al (1999) [43]	102	T: 28.7 ± 3.3 C: 27.7 ± 4.1	1) RN6 Qihai, RN3 Zhongji, RN4 Guanyuan, SP6 Sanyinjiao, SP4 Gongsun, LR3 Taichong, LR2 Xingjian, KI3 Taixi, KI1 Yongquan, PC6 Neiguan, HT7 Shenmen, EX EM12 Anmian, DU20 Baihui 2) BL23Shenshu, DU4 Mingmen, SP6 Sanyinjiao, SP4 Gongsun, LR3Taichong, LR2 Xingjian, KI3 Taixi, KI1 Yongquan, PC6 Neiguan, HT7 Shenmen, DU20 Baihui, EX EM12 Anmian	AC + EA, 30 min 1/day for 25 days, two month	The total treatment efficiency were 88.2% in the acupuncture-medicine combination group and 47.1% in the simple medicine group(*P* < 0.001)	Not mentioned

*Abbreviations:* *T* Trial group, *C* Control group, *yr* year, *AC* Acupuncture, *EA* Electroacupuncture, *WA* Warm acupuncture, *EPAS* mid-frequency electrical pulse acupoint stimulation, *IELT* Intravaginal Ejaculation Latency Time, *PEDT* Premature Effusion Diagnostic Tool, *CIPE-5* Chinese Index of Premature Ejaculation-5

## Discussion

### Features and advantages of acupuncture in PE

Acupuncture has evolved into a contemporary therapeutic approach with widespread international recognition [45, 46]. In the realm of male urological health, acupuncture has emerged as a noteworthy alternative or complementary therapeutic strategy for diseases like erectile dysfunction, premature ejaculation, and others [47–49]. Recent research has elucidated that acupuncture's influence on the body's meridian points triggers stimulation that navigates through the meridian system to impact the neuroendocrine network [50]. This stimulation, in turn, modulates the release of neurotransmitters and endocrine hormones. The intricate interplay within the meridian system helps the body maintain a relatively stable state, fostering the restoration of visceral functions—a fundamental aspect of disease prevention and treatment [51, 52].

Moreover, acupuncture is renowned for its stable and dependable therapeutic effects, with widely recognized long-term efficacy [53]. Sahin [36] demonstrated that a sustained regimen of acupuncture sessions can significantly prolong ejaculation latency and improve the sexual life satisfaction of couples. A prospective randomised controlled trial by Yang et al. [54]demonstrated that cognitive behavioural therapy (CBT) was significantly effective in improving ejaculatory control and sexual satisfaction. Compared to acupuncture, CBT focuses primarily on psychological aspects and may potentially overlook underlying physiological factors that contribute to PE. And CBT requires a longer period of psychological counselling, relying heavily on the therapist's expertise and the patient's cooperation. In contrast, acupuncture offers a more direct and shorter treatment duration, with better patient compliance. Furthermore, in clinical practice, acupuncture is generally associated with minimal adverse effects and is well-tolerated by patients with PE [55]. This provides a safer alternative for patients who are reluctant to try other therapies due to concerns about side effects associated with medication. The sustained efficacy of acupuncture can also contribute to reducing drug dependency and mitigating potential medication-related side effects cost-effectively [56].

### Characteristics of acupoints and meridians

The essence of acupuncture resides in the meticulous selection of acupoints and meridians for the treatment of PE. Acupoints represent specific locations along meridians, channels through which the flow of qi and blood enters and exits the body. As a result, these acupoints serve not only as reflective areas of diseases but also as focal points for therapeutic intervention, encapsulating the principle of "where the acupoints are, where the indications are." [53].

Acupuncture for the treatment of PE predominantly targets the foot-taiyin spleen meridian, foot-jueyin liver meridian, and foot-shaoyin kidney meridian in the selected studies, focusing on the utilization of proximal acupoints in the abdominal and lower limb regions. Among the frequently utilized acupoints are SP6 (Sanyinjiao), CV3 (Zhongji), LR3 (Taichong), and RN4 (Guanyuan). In traditional Chinese medicine, meridians are considered subservient to the viscera, extending beyond limbs and joints to serve as channels connecting the body's surface and the viscera [57]. SP6 (SanYinjiao), where the liver, spleen, and kidney meridians converge, corresponds to the lumbosacral medullary segmental innervation area [58]. Disorders of the spleen meridian, influencing qi and blood, can impact the Chong and Ren meridians, thus affecting reproductive function [59]. The liver meridian courses around the penile region, and is closely connected to the reproductive system, making it a specific acupoint for gynecological and andrological concerns [60, 61]. Zhang's study [62] on benign prostatic hyperplasia (BPH) indicated that warm acupuncture at SP6 (SanYinjiao) led to increased serum testosterone levels, suggesting a potential role in regulating sex hormones for PE treatment. RN4 (Guanyuan) [63] connects to the essence chamber in men, which refers to the anatomical region housing the vas deferens, seminal vesicle glands, and prostate glands, as described in modern medicine [64]. Acupuncture at RN4 (Guanyuan) has been confirmed that will cause elevated serotonin levels in humans [65]; and experiments in mice demonstrated an impact on sex hormone secretion, including serum testosterone, after acupuncture at RN4 (Guanyuan) [66]. CV3 (Zhongji), located along the abdomen's midline, plays a role in regulating qi flow within the essence chamber [67]. Animal research by Huang indicated that electroacupuncture at CV3 (Zhongji) significantly prolonged ejaculation latency in male rats, possibly linked to elevated serum serotonin levels [68]. LR3 (Taichong), positioned on the dorsal side of the foot, has been shown to activate brain regions associated with regulating negative emotions [69, 70]. Given the common comorbidity of anxiety or depression in PE patients, acupuncture at LR3 (Taichong) can effectively modulate qi movement and alleviate emotional distress. Also, a combination of multiple acupoints, along with appropriate techniques, is often employed to achieve a synergistic effect [71].

### Intervention

According to the selected studies, various acupuncture techniques were compared with the drug-only group, placebo group, and sham acupuncture group. Electroacupuncture [72] is an approach that combines traditional acupuncture with diverse forms of electrical current stimulation. Low-frequency electroacupuncture typically refers to an output frequency lower than 30 Hz, while high-frequency electroacupuncture ranges between 30HZ-1000HZ, with the upper limit generally not exceeding 1000 Hz [73]. Different frequencies and current intensities had different effects on the effectiveness of PE treatment. Some studies [74, 75] have suggested that under low-frequency (2 Hz) electroacupuncture, multiple stimulations are more likely to produce a cumulative effect. Additionally, compared to 100 Hz electroacupuncture, the therapeutic benefits are more enduring and effective. Lu's [37] research employed electrical stimulation of acupoints within a frequency range of 2–100 Hz. In contrast, Tang's [40] study emphasized mid-frequency electrical stimulation at acupoints without specifying the frequency and current intensity. He's [43] investigation, on the other hand, solely used electrical impulses to stimulate the RN3(Zhongji) and BL23 (Shenshu), with other acupoints treated using standard acupuncture. Many studies suggest that acupuncture has some placebo effect [76, 77], as shown by the lack of significant difference in efficacy between the acupuncture and sham acupuncture groups in randomized controlled trials. Sunay’s [38], Sahin’s [36], and Wang’s [42] studies used sham acupucture as the control group, have shown significant differences between the acupuncture and sham acupuncture groups, and the post-treatment effect was significantly higher than the baseline level. These differences suggest that acupuncture has effects beyond a simple placebo response. We argue that the mechanism of acupuncture treatment is complex and inevitably there will be some placebo effect, but not just a placebo. We need to look at it dialectically, acknowledging the existence of the placebo effect while also recognizing the inherent value of acupuncture.

### Retention time

The optimal amount of stimulation in acupuncture treatment is a critical determinant of efficacy [78, 79]. A retention time that is too short may fail to achieve the maximum peak of stimulation volume, while an excessively long retention time could lead to acupuncture tolerance and acupoint fatigue. There exists an optimal duration of needle retention, before which the relationship between time and efficacy is proportional, but beyond which the effect no longer increases with retention time, and may even decrease [80]. Lou [81], in the context of long-stay acupuncture at the DU20 (Baihui), discovered higher levels of 5-hydroxytryptamine(5-HT) in the hypothalamus and dopamine in rats compared to the ordinary acupuncture group, indicating that nerves are in a constant state under stimulation.

In the studies included above, the observation time for single needle retention ranged from 4 to 30 min, and it was observed that the efficacy of needle retention was superior within the 20–30 min range. Furthermore, the therapeutic effects on PE showed gradual improvement with an extended course of acupuncture treatment. Techniques involving continuous current stimulation in electroacupuncture and heat delivery in warm acupuncture are more likely to sustain effective nerve stimulation and achieve the sensation of "de qi"(means a feeling of soreness, numbness, distension, and heaviness after needling an acupuncture point).

### Probable neurobiological mechanisms

The neurobiological mechanisms of PE involve complex interactions of multiple neurotransmitters and brain regions [82]. Control of ejaculation is mediated through different parts of the limbic system, hypothalamus, amygdala, and thalamus of the brain. Neuroimaging studies [83] have found that human ejaculation activates the midbrain transition zone, thalamus, and parietal cortex, which work together to process both sexually relevant stimuli and peripheral somatosensory stimuli to form an integrated control of ejaculation. Since the twentieth century, the pathophysiology of premature ejaculation has been thought to be mediated primarily by the central nervous system, with complex interactions of 5-HT, dopamine, oxytocinergic, endocrinological, genetic, and epigenetic factors also thought to be part of the mechanism [84]. However, most research has been done using animal models, differences in genetic and epigenetic regulatory mechanisms and pharmacokinetics between species make direct comparisons challenging. While animal models provide valuable insights, caution must be exercised in interpreting and applying these findings. Animal experiments are the basis for conducting human trials, which can help us to gain a deeper understanding of the mechanisms of PE and to identify more selective therapeutic targets.And this underscores the need for further studies directly involving human subjects. This article will further explore the probable neurobiological mechanisms of acupuncture for PE, based on current neurobiological studies and the human clinical trials of acupuncture for PE included in this review.

**Fig. 2:**
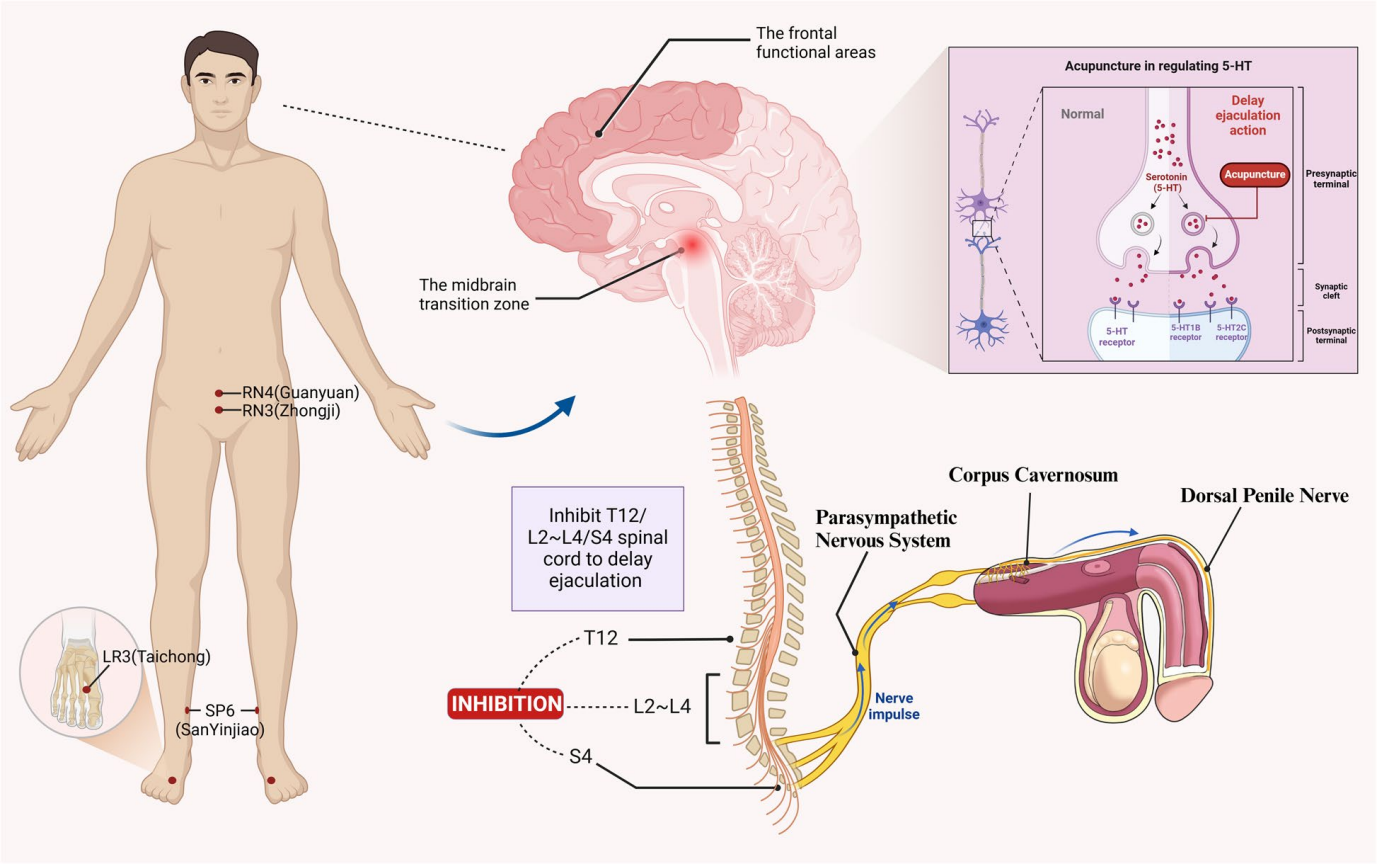
The location of the acupoints, and the neurobiological mechanisms by which these points prolong ejaculation time. Legend: The leftmost part of the figure shows the positioning of the most commonly used acupoints in the selected literature in the human body, and the exact location is highlighted with red dots; the middle part of the figure shows the brain and spinal cord centres, with distinct color representing the left frontal lobe (responsible for ejaculation control), and the midbrain (initiating ejaculation) is pointed out with a graduated red dot. The middle and lower section of the figure is the spinal cord centres controlling ejaculation, and acupuncture controls ejaculation by inhibiting the spinal cord segments. On the upper right side, the figure expounds upon the biological mechanisms involved (before and after acupuncture), while the lower right corner depicts nerve impulses (blue arrows) originating from the spinal cord and transmitting to penile nerves, regulating the corpus cavernosum, thereby inhibiting ejaculation.(The figure was created with biorender.com)

Acupuncture may exert its inhibitory effect on ejaculation by stimulating the left frontal functional areas. In healthy men, ejaculatory behavior involves the activation of several brain regions, including the midbrain transition zone, insula, cingulate, hypothalamus, amygdala, occipitotemporal lobe, and frontal lobe [85, 86]. The midbrain transition zone is particularly vital in initiating ejaculation [87, 88]. When men are sexually stimulated, the midbrain transition zone is activated first (Fig. 2), followed by the activation of cortical regions [89]. Notably, these activated cortical regions are predominantly located in the right hemisphere of the brain and include the posterior portion of the right insula, the deep regions of the dorsal posterior temporal lobe, the central portion of the midbrain, the medial segments of the pallidal globes on both sides, as well as the deep nucleus of the cerebellum and cerebellar vermis. In contrast, the inactivated brain regions tend to be concentrated in the left hemisphere [90]. It has been observed that patients with PE exhibit thicker cortex in various brain systems, including the frontal, parietal, occipital, and limbic regions compared to healthy controls [91]. Additionally, PEDT scores are negatively correlated with cortical thickness in the left superior frontal middle gyrus [92]. Some researchers have also identified abnormal function of the left inferior frontal gyrus as the primary cause of reduced control over ejaculation time in patients with primary premature ejaculation [93]. Earlier neuroimaging studies have demonstrated that stimulating different acupoints can activate distinct brain regions and exert varying effects on the central nervous system. For instance, Zhang's [94] functional magnetic resonance imaging study on 11 subjects revealed that acupuncture at SP6 (SanYinjiao), LI4 (Hegu), ST36 (Zusanli), and PC6 (Neiguan) resulted in increased average signal intensity in the bilateral inferior frontal gyrus region. Similarly, Yang's [95] clinical study found that acupuncture at SP6 (SanYinjiao), LR3 (Taichong), and ST36 (Zusanli) led to varied levels of activation in functional brain areas such as the bilateral inferior frontal gyrus and cerebellar regions. Moreover, a study [96] investigated changes in brain activation during acupuncture at the LR3 (Taichong) in healthy individuals of different genders and found that acupuncture at this acupoint significantly activated brain regions, especially the bilateral frontal lobe, compared to the sham acupuncture group. Consequently, nerve impulses generated by acupuncture can traverse the spinal cord, activating the functional areas of the left frontal lobe (Fig. 2). This process enhances the ability to control ejaculation, ultimately prolonging the latency time in patients with PE. Moreover, distinct acupoints may stimulate the activation of the same brain functional area, explaining the synergistic effects produced by the combined use of multiple acupoints [94]. However, it is crucial to acknowledge that the degree of activation of the same acupoints may vary among different individuals, possibly owing to differences in their physical attributes. In addition, PE is also affected by psychological and social factors, which interact with each other to influence the course of the disease and its treatment. Therefore, more rigorous experiments are still necessary to further substantiate these findings and to verify the neurobiological mechanisms of acupuncture for PE.

Acupuncture potentially extends ejaculation duration by dampening the excitability of spinal cord centers. Within the comprehensive sexual response cycle encompassing semen secretion and ejaculation, the vegetative nerves govern semen production, allowing for relatively controllable timing [97]. The intricate neurophysiological network regulating PE through acupuncture points involves diverse distributions across various meridians and body parts, yet these points maintain a significant connection with PE. For instance, SP6 (SanYinjiao) is predominantly associated with the saphenous nerve and tibial nerve [98]. The saphenous nerve innervates the superficial skin and fascia, originating from spinal cord segments L2-L4 (Fig. 2), where the L2 ganglion controls semen secretion activity. Meanwhile, the tibial nerve, originating from segment S4, governs ejaculatory activity [99]. A study utilized the horseradish peroxidase-conjugated cholera toxin B subunit (CB-HRP) tracing technique to examine the relationship between acupuncture at RN4(Guanyuan) and ganglion segments, and found positive neurons distributed in the T11-L3 ganglion segments. After electroacupuncture treatment, the peak stage of positive neurons shifted up to the L2, implying that the L2 ganglion segments may serve as the primary pathway for the signal transmission from acupuncture at RN4 (Guanyuan) into the spinal cord [100]. On the other hand, the CV3 (Zhongji) was attributed to the T12 ganglion segment, overlapping with the semen secretion center [101]. All the aforementioned studies indirectly demonstrate the close neurobiological relationship between CV3 (Zhongji), RN4 (Guanyuan), and the related spinal cord centers. This connection provides a neurobiological basis for acupuncture treatment of PE within the innervation zones of these neural segments.

Acupuncture may elevate the ejaculation threshold by regulating disorders in the neurotransmitter 5-HT, thereby delaying ejaculation. In contemporary medicine, premature ejaculation is associated with a weakened inhibitory process in the cerebral cortex and heightened excitability in the higher sexual centers. The neurotransmitter 5-HT plays a crucial role in the central nervous system, distributed widely in the hypothalamus, brainstem, and spinal cord, participating in the regulation of the ejaculatory process [102]. Dysfunctional 5-HT, with its rapid uptake, leads to a decrease in 5-HT concentration, resulting in a shorter ejaculatory latency. To exert its biological effects, 5-HT must bind to its corresponding receptor [103]. Animal studies [104] have identified three subtypes related to ejaculation regulation: activation of 5-HT1B and 5-HT2C receptors prolongs the latency to ejaculation, while stimulation of 5-HT1A receptors shortens the latency to ejaculation. Studies [105] demonstrated that electroacupuncture at SP6 (SanYinjiao) and ST36 (Zusanli) could regulate hypothalamic and serum 5-HT concentration and expression in tissues. Notably, ST36 (Zusanli) was found to be significantly more effective than SP6 (SanYinjiao). Additionally, electroacupuncture at DU20 (Baihui) and EX-HN3 (Yintang) was shown to up-regulate 5-HT1B receptor mRNA expression, while electroacupuncture at LI4 (Hegu), PC6 (Neiguan), and ST36 (Zusanli) acupoints down-regulated the excitability of 5-HT1A receptors in the spinal cord [106, 107]. These findings suggest that acupuncture may enhance the excitability of 5-HT1B receptors and decrease the excitability of 5-HT1A receptors by modulating 5-HT concentration, ultimately raising the ejaculation threshold and prolonging ejaculation latency (Fig. 2).

Acupuncture may prolong ejaculation by reducing the sensitivity of sensory nerves around the penile head or inhibiting rhythmic muscle contractions. Anatomically, the lower abdomen and lumbosacral region contain abundant pelvic plexus nerves, sharing the same spinal cord segments as nerves innervating the genitals, bladder, and urethra. Acupuncture can effectively stimulate these pelvic plexus nerves [108, 109]. The acupoints most frequently used in the abdomen in this review are RN4 (Guanyuan) and RN6 (Qihai), which are in proximity to the penis. The benign stimulus impulses generated by needling these acupoints may have a higher likelihood of transmitting to the penis or perineum. Upon receiving these nerve impulses, the nerves within the pubic area could potentially slow down semen ejaculation by inhibiting rhythmic contractions of the bulbocavernosus and ischiocavernosus muscles (Fig. 2). However, this theoretical understanding is speculative, and currently, no studies have confirmed how acupuncture precisely acts on peripheral penile nerves to extend ejaculation.

Acupuncture may impact ejaculation by modulating hormone levels in men. Hormones play a pivotal role in ejaculatory control, and abnormal hormone levels may directly or indirectly affect ejaculation control. For example, testosterone and thyroid hormone levels can influence ejaculatory control in individuals with PE [110]. Studies [111, 112] have revealed a close relationship between the nitric oxide synthase-phosphodiesterase 5 type system and contractile function during ejaculation in men, which is influenced by testosterone levels, indicating that testosterone has a role in the ejaculation process. Research by Corona G [113] showed a negative correlation between serum testosterone levels and the development of PE, with higher serum testosterone levels associated with an increased likelihood of PE, and delayed ejaculation being more common in individuals with lower serum testosterone. Sakamoto H [114] also observed significantly higher serum-free testosterone and serum follicle-stimulating hormone levels in patients with PE compared to those without, although there was no notable difference in serum total testosterone levels between the two groups. Animal experiments [115] demonstrated that electroacupuncture stimulation of RN4 (Guanyuan) and SP6 (SanYinjiao) could regulate testosterone levels in rats, reducing serum testosterone in aged rats. Moreover, acupuncture at RN4 (Guanyuan) in mice increased the weight of gonadal organs (testes and spermathecae) and improved sexual function. Zheng [116] reported a decrease in serum testosterone levels after electroacupuncture at the RN3 (Zhongji).

### Limitations

This review encompasses eight studies that meticulously adhere to the search strategy outlined in our article. This paper aims to scrutinize the role of acupuncture in individuals with premature ejaculation from the perspective of traditional Chinese medicine and its associated mechanisms, providing an initial foundation for the utilization of acupuncture in premature ejaculation treatment. One primary limitation lies in the variability across study methodologies and acupuncture protocols employed in the existing literature, and the lack of standardization prevents us from drawing universally applicable conclusions. Variations in needling techniques, acupoint selection, and treatment duration pose challenges in synthesizing results across different studies. Another limitation is the small sample size, thereby restricting our ability to comprehensively interpret the mechanisms of acupuncture in treating PE. Addressing these constraints will be pivotal in advancing our understanding of acupuncture's therapeutic potential and refining its clinical applications for managing premature ejaculation. The discussions on the mechanisms related to acupuncture for premature ejaculation may be limited due to insufficient evidence and warrant further exploration.

## Conclusion

This review offers valuable evidence and guidance for clinicians treating PE through acupuncture. In comparison to SSRIs, acupuncture did not demonstrate a significant advantage in improving IELT and PEDT scores. However, a noteworthy improvement was observed when compared to sham acupuncture or when used in combination with medication. Under traditional Chinese medicine theory, the efficacy of acupuncture is influenced by factors such as location, retention time, and treatment cycle. Based on our findings, we posit that acupoints in the abdomen and bilateral lower limbs, primarily along the Ren and Spleen meridians, such as CV4 (Guanyuan), SP6 (SanYinjiao), and ST36 (Zusanli), are particularly suitable for treating PE in clinical practice. The primary mechanism of acupuncture for addressing PE appears to involve symptom amelioration through the modulation of the nervous and endocrine systems, although these mechanisms warrant further validation. Subsequent research efforts should prioritize more standardized designs, encompassing treatment duration, retention time, and selected acupoints to the development of scientifically grounded clinical treatment protocols. The role of the placebo effect also needs to be clarified to determine the specific ways in which acupuncture exerts its therapeutic effects.

## Data Availability

No datasets were generated or analysed during the current study.

## Abbreviations

AC: Acupuncture
BPH: Benign Prostatic Hyperplasia
C: Control Group
CB-HRP: Horseradish Peroxidase-Conjugated Cholera Toxin B Subunit
CBT: Cognitive Behavioural Therapy
CIPE-5: Chinese Index of Premature Ejaculation-5
EA: Electroacupuncture
ED: Erectile Dysfunction
EPAS: Electrical Pulse Acupoint Stimulation
IELT: Intravaginal Ejaculation Latency Time
IIEF: International Index of Erectile Function
ISSM: International Society for Sexual Medicine
PE: Premature Ejaculation
PEDT: Premature Effusion Diagnostic Tool
PSSD: Post-SSRI Sexual Dysfunction
SSRIs: Selective Serotonin Reuptake Inhibitors
T: Trial Group
TCM: Traditional Chinese Medicine
WA: Warm Acupuncture
Yr: Year
5-HT: 5-Hydroxytryptamine

## Availability of data and materials

All data generated or analyzed during this study are included in this published article.
